# The SUCCESS Peer Mentoring Program for College Students with Concussion: Preliminary Results of a Mobile Technology Delivered Intervention

**DOI:** 10.3390/ijerph20085438

**Published:** 2023-04-07

**Authors:** Katy H. O’Brien, Yalian Pei, Amy M. Kemp, Rebecca Gartell, Russell K. Gore, Tracey Wallace

**Affiliations:** 1 Department of Communication Sciences and Special Education, University of Georgia, Athens, GA 30602, USA; 2 Courage Kenny Rehabilitation Institute, Allina Health, Minneapolis, MN 55407, USA; 3 Virginia C. Crawford Research Institute, Shepherd Center, Atlanta, GA 30309, USAtracey.wallace@shepherd.org (T.W.); 4 Complex Concussion Clinic, Shepherd Center, Atlanta, GA 30309, USA

**Keywords:** brain injuries, concussion, students, rehabilitation, peer support, technology, mHealth, smartphone

## Abstract

Concussions are caused by a hit or blow to the head that alters normal brain functioning. The Success in College after Concussion with Effective Student Supports (SUCCESS) program was developed to provide students with psychosocial support and resources—both key components of concussion management—to assist in recovery and return-to-learn following concussion. In this preliminary evaluation of intervention efficacy, SUCCESS was delivered through a mobile application connecting mentors (students who have recovered from concussion and successfully returned to school) with mentees who were currently recovering. Mentor–mentee pairs met virtually through the app, using chat and videoconferencing features to share support, resources, and program-specific educational materials. Results from 16 mentoring pairs showed that mentee symptoms (V = 119, *p* = 0.009) and academic problems decreased (V = 114.5, *p* = 0.002), while academic self-efficacy increased (V = 13.5, *p* = 0.009) following mentoring. As expected, mentor measures were stable, indicating that providing mentoring did not exacerbate previously resolved concussion complaints. Virtual peer mentoring provided through a mobile application may be a feasible intervention to support academic success and psychosocial processing during recovery for college students with concussion.

## 1. Introduction

Millions of concussions, a type of traumatic brain injury (TBI), are sustained annually in the United States [1]. Although awareness of the effect and risk of concussion associated with athletic participation has risen dramatically over the last 10 years, the incidence of concussion on college campuses is higher in non-athletes than athletes, so that 1 in every 80 students may be expected to experience a concussion each school year [2]. In addition, between 16% and 28% of college students report a history of concussion [3,4], increasing risk for complicated or prolonged recovery [5]. Concussions may be particularly problematic for college students given the high stakes and limited support within the learning environment [6], thus necessitating aggressive care to mitigate short- and long-term consequences. Although recovery data on the general postsecondary student population are scarce, younger students often need up to a month or more to recover [7], and guidelines for adults suggest similar timeframes [8]. Similarly, 20 to 30% of younger students may show lengthier recovery trajectories [9], and some studies suggest that a majority of adults with mild TBI continue to experience symptoms at 6 and 12 months that disrupt daily functioning, with memory problems and slowed thinking being particularly problematic [10,11]. In studies specific to college-age students, some have found resolution in as few as 18 days in smaller samples [12], or that incomplete recovery at one month post-injury was associated with risk factors, such as being female or having a history of concussion [13]. One study comparing collegiate athletes with a general college student sample found that non-athlete students were more likely to have complicating factors at baseline as well as greater symptomatology and poorer performance on neurocognitive assessments of attention during initial post-injury assessment [14], suggesting heightened risk for prolonged recovery.

Post-concussion symptomatology puts students at risk for loss of educational inclusion, directly impacting academic success, educational confidence, and quality of life, and can have financial repercussions in loss of scholarships, work, and extended time to graduation [13,15,16]. Specifically, concussion symptoms may include inattention, headache, sensitivity to noise/light, memory problems, and/or emotional symptoms such as anxiety, making academic participation difficult (e.g., paying attention in class, memorizing formulas or terminology, keeping up with and taking notes during lectures). Math and science in particular have been identified as difficult for students following concussion [12], but many students experience difficulty with reading [17,18], impacting academic performance and exacerbating symptoms across subject areas. Students also often feel misunderstood when approaching professors to discuss their needs [15].

Recent timelines of concussion recovery suggest athletes recover more quickly than the general population, and needs in varsity, school-sanctioned sports programs are tightly managed by laws and procedures. In contrast, athletes participating at other levels—such as club or intramural sports—as well as non-athletes are a neglected clinical population, yet these students are at the pinnacle of their learning, along with developing independent social and professional networks. Non-varsity athletes also tend to have more cognitive and persistent problems than their varsity athlete peers [14], increasing the likelihood of academic problems interfering with successful advancement in their studies.

### 1.1. Peer Mentoring in a Neurobiopsychosocial Model of Concussion

Students with concussion exist in a space where they need medical and educational support, yet students may be unsure about how to seek care and injury effects make it unlikely that they can find appropriate resources on their own. In a survey of college athlete attitudes toward concussion reporting, students were far more likely to report an injury to a teammate/peer compared with a coach or athletic trainer [19]. Peer mentoring has been shown to be an effective tool to help people access resources, cope with challenges, and learn to manage their condition [20]. In both developmental and acquired disability populations, people who engage in peer mentoring have demonstrated improvement in mood, behavioral control, and coping [21,22,23,24]. Mentoring also fosters the development of strong social supports and resilience [25], which has in turn been associated with positive outcomes following TBI [26]. Mentors often also benefit from the experience, building self-efficacy and confidence in their own abilities [27], and they feel prepared to face and overcome challenges presented by meeting new peers and collaboratively problem-solving needs [28].

In addition, peer-led education, as compared that provided by healthcare professionals, has also resulted in increased adherence to health recommendations and greater self-efficacy for disability management [29]. Such increases in self-efficacy have been observed in a range of studies examining self-management of chronic disease processes [30,31], and have been found to reduce re-hospitalization as well [32]. Similarly, studies examining knowledge outcomes following peer-led education in athletic contexts to improve reporting of concussion or understanding of concussion effects [33] have shown that athletes demonstrate gains in both knowledge and attitudes around concussion reporting [34]. Although one study is underway examining peer-support during recovery for high school students with concussion [35], research into peer-mentoring as an intervention approach for management of concussion has not yet been adequately investigated, and no digital solutions exist to meet needs of this population.

**Figure 1:**
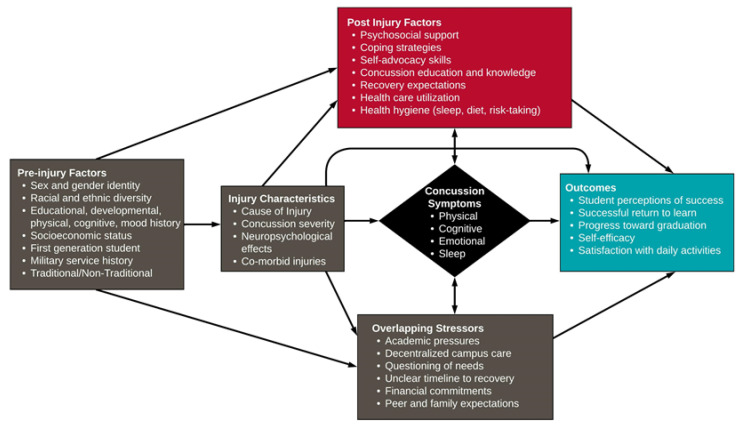
Neurobiopsychosocial model of concussion, adapted to reflect the needs and experiences of postsecondary students. SUCCESS targets modifiable post-injury factors in red, and is proposed to result in improved concussion, academic, and psychosocial outcomes shown in blue (adapted with permission from Yeates, 2010 [36] and from Polinder et al., 2018 [37] through the Creative Commons Attribution License).

Current models of concussion describe a neurobiopsychosocial injury [36,37,38] with baseline student characteristics interacting with the injury, systems of care, and the student’s psycho-emotional processing of their experiences. As depicted in gray boxes in Figure 1, many elements of this model are not modifiable at the point of injury. In contrast, post-injury factors around accessing available care, understanding concussion, and managing psychological responses are modifiable (shown in red). In addition, support provided by peers, family, and those with similar experiences of concussion has been found to be particularly meaningful to students following injury [39]. Individuals who receive appropriate education about management techniques and feel more supported post-concussion experience fewer symptoms and recover more quickly [7,40]. Peer mentoring targets these modifiable aspects of the neurobiopsychosocial model of concussion, directly addressing how students understand, think about, and act on their injury experiences. 

College students with concussion may particularly benefit from a peer mentoring approach. Similar to more severely impaired student-aged survivors of TBI, peer mentoring directly addresses Belch’s (2004) key elements for success of students with disabilities in higher education: establishing feelings of belonging, involvement, and purpose [41]. Mentoring allows a more experienced peer to provide social support and share knowledge with a less experienced peer (see [22] for review of peer mentoring after TBI). Because pathways to concussion care vary across campuses, mentors who have navigated those same paths while recovering from concussion may be well suited for providing context specific guidance around supports available or challenges that may be encountered. Mentors can also serve as role models or tangible examples of recovery and a successful return to learn and daily activities [32]. Studies of peer-led education show that peers like talking to other peers as well, often reaching out to peers before medical, sport, or educational professionals [34,39]. Serving as a mentor builds interpersonal skills and confidence that can translate to solving problems beyond college as well [27,40].

### 1.2. mHealth Peer Mentoring

Although so called screen time—or time spent viewing computers, smartphones, tablets, televisions, or other devices—is often associated with symptom provocation following concussion, recent studies have shown that screen time appears to impact recovery and symptoms most during the first two to four days post-injury [42]. This means that although some people may experience sensitivity to screens and light during their recovery, use of screens beyond the period immediately following injury is not expected to negatively impact recovery. In fact, several studies have examined the use of teletherapy and videoconferencing in individuals with concussion, finding interventions to be equally effective regardless of delivery modality [43,44] or even perform above and beyond usual care [45,46]. In a study examining the experiences of children with concussion and their parents, a key finding was that the children and young adults preferred videoconferencing and video formats, and also wanted to connect with peers to learn directly from people who had been through similar experiences. In contrast, parents did not share the same interest in videos and videoconferencing interactions [47]. 

The aim of the current report was to conduct preliminary testing of a peer mentoring program for college students with concussion that was delivered through an mHealth mobile application: Success in College after Concussion with Effective Student Supports (SUCCESS). SUCCESS was developed with the input of students with concussion, educators, parents of students with concussion, and patient advocates, along with medical, rehabilitation, and disability service professionals, to meet student needs after concussion [48,49]. SUCCESS pairs newly injured college students with students who have sustained concussions, successfully recovered, and returned to school. Peer mentors link students to resources that support recovery and, critically, facilitate a circle of communication with mentees to build a community of support. The program was delivered virtually, through a mobile application (“app”) (www.peersuccess.org/app (accessed on 1 February 2023)). In addition to facilitating virtual videoconferencing meetings, the app provided students access to educational materials about concussion [50]. The research questions for this study were as follows: (1) Do college students with a history of concussion participating as mentees and mentors in the SUCCESS program demonstrate changes on measures of concussion symptoms, academic performance, and psychosocial health after mentoring? (2) Does participant feedback on the SUCCESS program indicate program acceptability by mentees and mentors? It was hypothesized that mentees would experience reduced symptoms, stable academic experiences, and improved quality of life and self-efficacy following mentoring in the SUCCESS program. Mentors were hypothesized to experience stable symptoms and academic performance, with similar gains to psychosocial measures as their peer mentees. It was also hypothesized that participant feedback would lead to the identification of opportunities to improve program design but would indicate general program acceptability.

## 2. Materials and Methods

### 2.1. Participants

Participants included two groups: (1) student mentors who had recovered from concussion, maintained academic standing, and were at least 6 months post-injury; and (2) student mentees currently recovering from concussion. Both undergraduates and graduate students were eligible to participate as either mentors or mentees if they sustained a medically diagnosed concussion while enrolled as a student, were over the age of 18, were able to speak and read English, and did not have history of more severe traumatic brain injury or other neurological disorder. Previous history of concussion was acceptable. All research activities were reviewed and approved by both the University of Georgia and Shepherd Center’s Institutional Review Boards. Mentors and mentees were recruited through a specialty concussion clinic and a university email listserv. Sixteen mentor and mentee pairs consented, completed pre-measures, and participated in intervention activities during an academic semester (see Table 1 for participant characteristics).

### 2.2. Intervention

The SUCCESS program includes: (1) mentor training, (2) mentee-driven matching, (3) educational resources, and (4) in-person and in-app mentoring.

#### 2.2.1. Mentor Training

**Figure 2:**
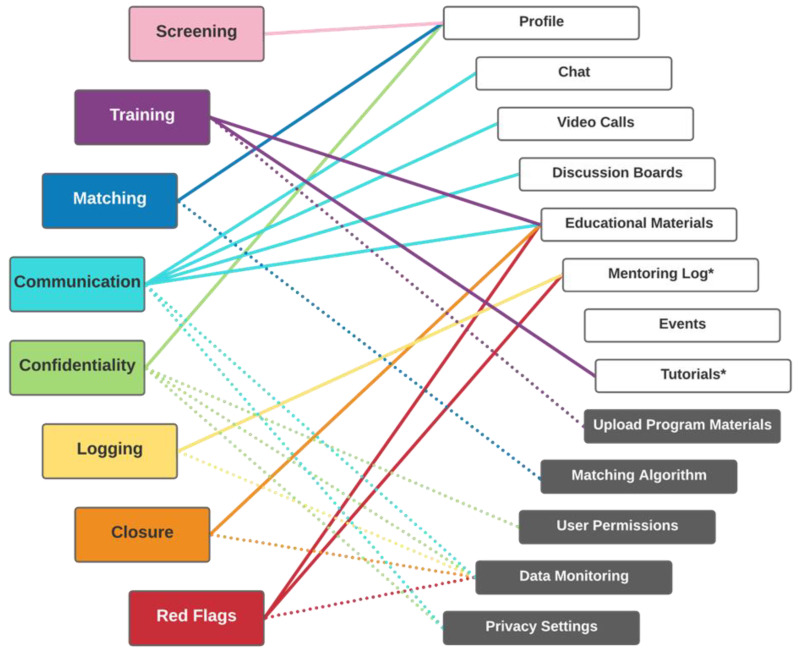
Mapping of key peer mentoring program components to platform features. Key components of peer mentoring programs (colored boxes) are supported by app features (white boxes, solid lines) and administrator portal functions (gray boxes, dotted lines). * Features not native to the app, but that were able to be addressed by linking to external resources, such as materials or surveys.

The program was anchored in an accessible (e.g., 508-compliant) mobile app that included the training for mentors, matching metrics, educational resources, text and videoconferencing for communication, and reminders for mentoring meetings. The Qooper platform was selected for mHealth delivery based on fit with key components of peer mentoring programs identified during discovery [48]. The platform includes both an administrator portal to manage and view mentor and mentee interactions, along with the mobile app that is compatible with both Android and iOS operating systems. Figure 2 describes matching of key components to platform features. 

Potential mentors completed a two-hour online training on concussion, college, coping, and roles/responsibilities of mentors, mentees, and the SUCCESS team. Mentors read text and watched videos of students with concussions sharing their experiences, and then responded to closed and open-ended questions. To be selected, mentors must have achieved at least 80% proficiency on multiple choice questions. Research assistants (RAs) also reviewed free text responses to ensure mentors demonstrated the ability to take the mentee’s perspective and incorporate training material in guidance they might provide. Selected mentors completed a second 40 min online training explaining program procedures, use of the app, and when and how to contact the SUCCESS team (see Table S1 in Supplemental Materials for training outlines).

#### 2.2.2. Matching

Student mentees completed profiles in the app detailing relevant background information, concussion experiences, needs, and preferences for matching. Mentees ranked their preferred order of matching, including level in school, major, sports participation, gender, and symptom profiles. RAs communicated with student pairs twice during the first week to ensure contact had been made and both parties wanted to continue with the match. All pairs were matched for at least 4 weeks, and could opt in to being paired for up to 8 weeks.

#### 2.2.3. Educational Resources

Resources were based on student preferences and literature on student needs following concussion [49,50]. Topics included (a) Concussion Basics; (b) Return-to-Learn Part 1: Accommodations, Advocacy and Resources; (c) Return-to-Learn Part 2: Study Skills and Learning Strategies; and (d) Returning to Activity (see Figure S1 in Supplementary Materials).

#### 2.2.4. In-App Mentoring

**Figure 3:**
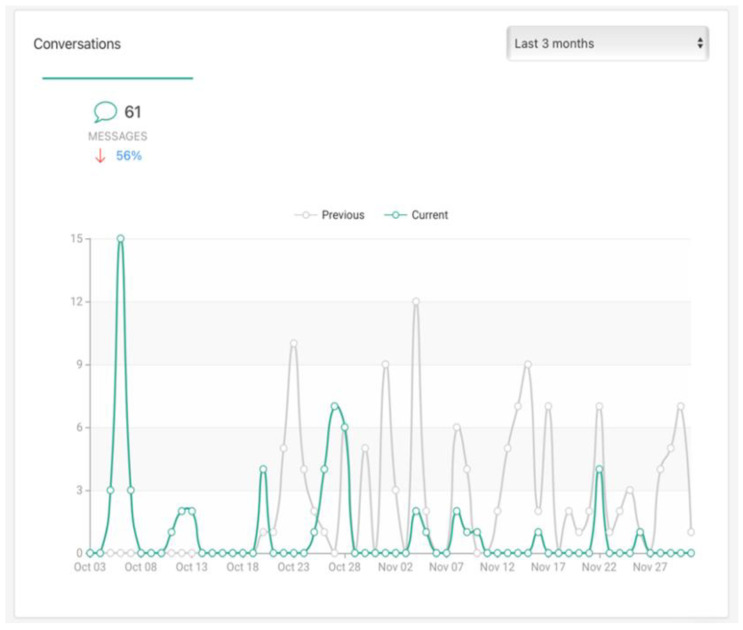
Screenshot of sample data monitoring of peer contact. “Current” reflects the most recent time period, and “Previous” the same time interval prior to the most recent measurement period.

Student pairs met weekly through videoconferencing or chat meetings in the app. All app-based communication was automatically tracked and available in the administrative portal (see Figure 3).

### 2.3. Measures

Participants completed all survey measures pre- and post-mentoring intervention. Both mentees and mentors completed measures that examined concussion symptoms, general self-efficacy, and mental health. In addition, measures that addressed academic dysfunction, academic self-efficacy, and quality of life were administered to mentees. After mentoring was complete, mentees completed a program feedback survey describing utility of the program and likelihood of recommending SUCCESS to other students with concussion, in either the role of mentor or mentee (see Table 2 for detailed measure descriptions).

### 2.4. Data Analysis

To assess the impact of mentoring on mentors and mentees, Wilcoxon signed-rank tests were used to compare concussion symptoms, academic dysfunction, academic self-efficacy, general self-efficacy, mental health, and quality of life pre- and post-mentoring. Results of the mentee post-mentoring feedback survey sliding scale questions are presented descriptively, alongside summaries of open-ended responses.

## 3. Results

### 3.1. Mentees

#### 3.1.1. Concussion Symptoms

**Figure 4:**
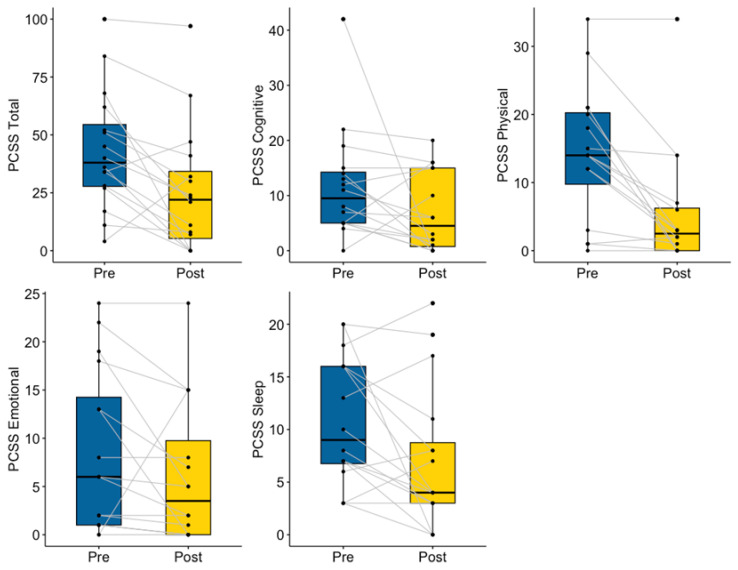
Mentee Pre- and Post-Mentoring Concussion Symptoms.

Concussion symptoms measured by the PCSS decreased significantly from pre- to post-mentoring (V = 119, *p* = 0.009), particularly in the physical domain (V = 103, *p* = 0.002), the sleep domain (V = 98.5, *p* = 0.031; see Figure 4), and affective domain (V = 66, *p* = 0.036). No significant differences were observed in cognition (V = 90.5, *p* = 0.088), although 12 of 16 participants reported experiencing fewer cognitive symptoms post-mentoring (see Figure 4).

#### 3.1.2. Academics

**Figure 5:**
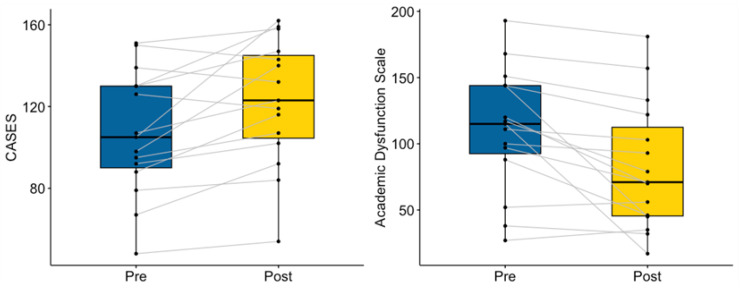
Mentee Pre- and Post-Mentoring Academic Function.

After mentoring, mentees reported significantly fewer academic difficulties, indicated by changes in ADS scores (V = 114.5, *p* = 0.002). Pre- and post-mentoring CASES scores demonstrated a significant increase in academic self-efficacy (V = 13.5, *p* = 0.009; see Figure 5).

#### 3.1.3. Psychosocial Function

**Figure 6:**
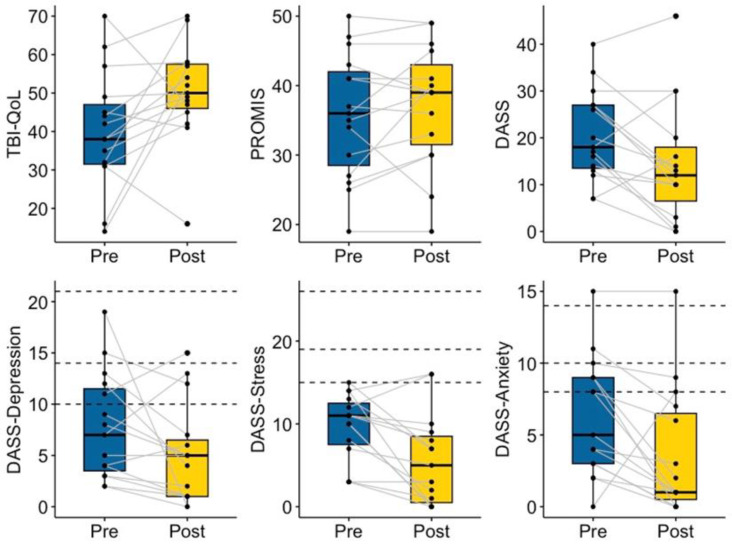
Mentee Pre- and Post-Mentoring Mental Health. Dotted lines indicate recommended cutoff scores for mild, moderate, and severe symptom presentations [54].

While change in TBI-QoL scores approached significance, 11 out of 15 mentees demonstrated increases in quality of life (V = 25, *p* = 0.050). A significant decrease in negative emotions (V = 87.5, *p* = 0.030) was demonstrated in pre–post-mentoring DASS scores, with significant decreases in anxiety (V = 92, *p* = 0.014) and stress (V = 93.5, *p* = 0.011). Although it did not reach statistical significance (V = 80, *p* = 0.089), eleven mentees reported decreased depressive symptoms. General self-efficacy measured by PROMIS was stable pre- and post-mentoring (V = 24.5, *p* = 0.271, see Figure 6). 

#### 3.1.4. Mentee Post-Intervention Feedback Survey

Based on post-mentoring feedback provided by 14 mentees, mentees gave the highest rankings to the helpfulness of the support provided by their mentors (M = 96.07, Mdn = 100) and reported feeling highly successful in communicating with and meeting their mentors. Mentees also felt that they were able to communicate easily with the SUCCESS team when needed (M = 81.08, Mdn = 80.5) and that overall, participating in SUCCESS was very helpful (M = 90, Mdn = 96). In contrast, mentees were more reserved if still positive in their rankings of helpfulness of the app (M = 62.89, Mdn = 60), even though they reported relatively high success in using the app. Table 3 provides a descriptive summary of program feedback response data. 

In open-ended questions, mentees provided positive feedback across areas, with the most common response being related to appreciation of having someone who understood their experience. For example, one mentee wrote, “I liked having someone to talk to who knew how I was feeling”. Similarly, another mentee said, “I thought it was very helpful in the sense of I finally felt like I was talking to someone who got it and didn’t feel like I was on my own or being misunderstood”. Several mentees specifically commented on mental health effects related to that feeling of being understood. One mentee shared, “It helped me emotionally because it was great having someone to talk to who knew how I felt and could help me through my situation”, while another wrote, “It helped my mental health and being able to talk to someone who was just like me. I didn’t feel alone”.

Regarding program drawbacks or recommendations toward modifying the program, comments were few, but one mentee had hoped to meet other mentors and mentees as well, and another commented that it might be tricky to find a good match for a mentee. Two mentees expressed concerns about the time commitment, with one summarizing his feelings as, “I wouldn’t call them problems, but one downside to the program, and I mean no offense, is that it is ‘just another thing to do’ if that makes sense”. Only one mentee provided a recommendation, suggesting that SUCCESS recruitment should occur as close to the time of injury as possible. This mentee described that she felt the program would have been even more helpful if she had been able to join at the time of her initial diagnosis. No feedback was provided related to the app or app usage.

### 3.2. Mentors

Among the 16 mentee–mentor pairs, two mentors were paired with more than one mentee, so only the first round of matching was included in the data analysis. Additionally, one mentor did not complete post-mentoring measures. Therefore, pre- and post-measures of ten mentors were included in the analyses.

#### 3.2.1. Concussion Symptoms

**Figure 7:**
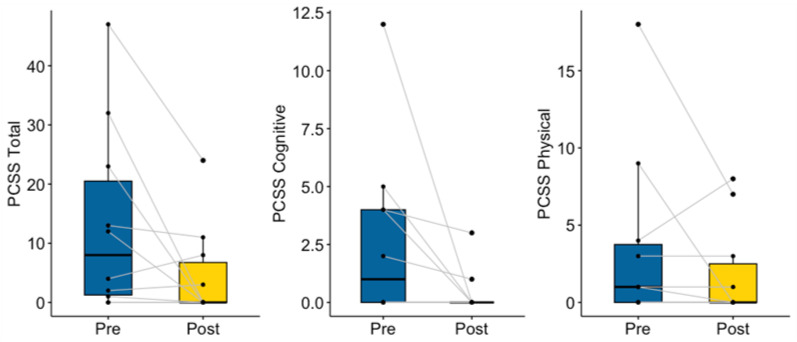

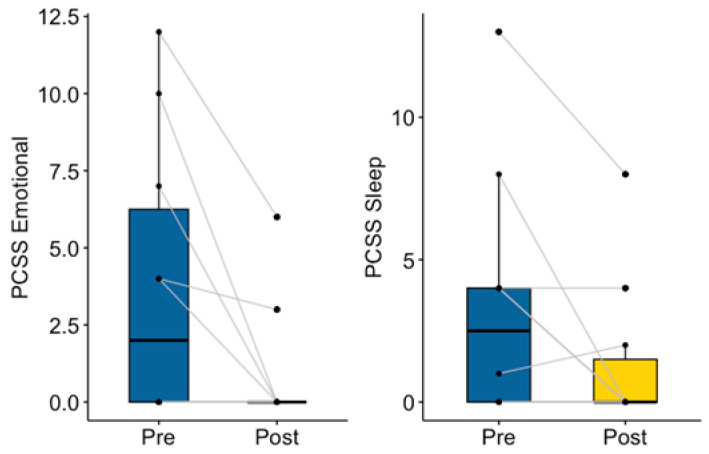
Mentor Pre- and Post-Mentoring Concussion Symptoms.

As expected, mentors did not show significant changes in PCSS scores between the two timepoints (V = 30.5, *p* = 0.09), which may also be explained by their low level of symptoms at baseline (see Figure 7). Specifically, two reported zero concussion symptoms in all four domains, five reported zero cognitive and emotional symptoms, and four reported zero physical and sleep symptoms pre-mentoring. Regardless of the low baselines, the number of mentors who reported zero concussion symptoms increased post-mentoring, including six mentors who reported zero concussion symptoms across domains, six reporting zero physical symptoms, seven reporting zero sleep symptoms, and eight reporting zero cognitive or emotional symptoms. Overall, concussion symptom levels were maintained from pre- to post-mentoring, suggesting engagement in mentoring did not aggravate previously resolved concussion symptoms.

#### 3.2.2. Psychosocial Function

**Figure 8:**
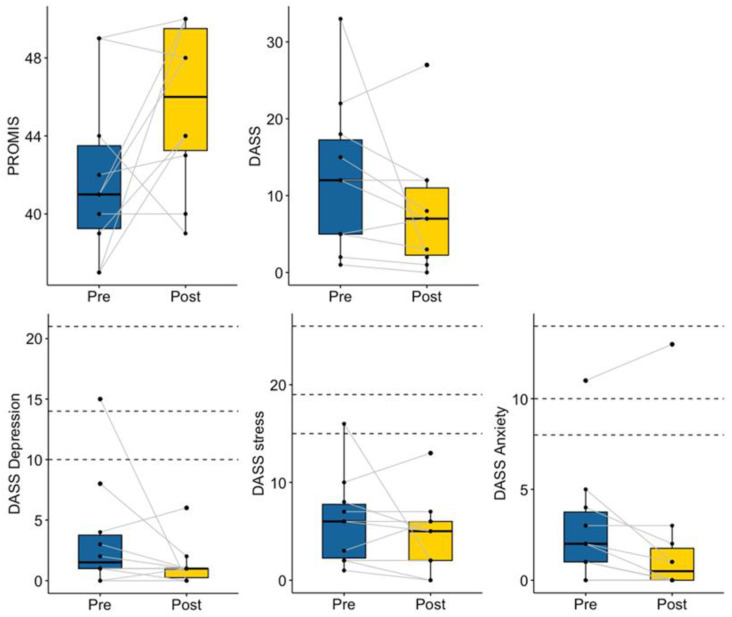
Mentor Pre- and Post-Mentoring Mental Health. Dotted lines indicate recommended cutoff scores for mild, moderate, and severe symptom presentations [54].

Although the change was not statistically significant (V = 36, *p* = 0.12), seven out of ten mentors showed a decrease in negative emotions measured by the DASS. More importantly, one mentor with moderate levels of depression and mild levels of stress pre-mentoring reported both to be within normal limits upon completion of mentoring, suggesting clinical significance. In contrast, the single participant with elevated levels of anxiety at baseline experienced an increase in those symptoms. In addition, mentors showed an increasing trend in general for self-efficacy (V = 6.5, *p* = 0.06; see Figure 8), with seven of ten participants demonstrating gains from pre- to post-mentoring.

#### 3.2.3. Mentor Post-Intervention Feedback Survey

Mentors rated the program highly overall, and were very likely to recommend it to either acutely injured or recovered students (see Table 4). They gave particularly high ratings to the training and the SUCCESS team, and gave positive but lower ratings to the program handouts and the mobile app. Mentors provided more written feedback in open-ended questions than did mentees, although it reflected much of the same feedback of that group.

In response to open-ended feedback questions, mentors reacted positively to both the SUCCESS program and the app, with most feedback directed toward improving the app. Mentors described the program itself as being well-structured and that overall they felt prepared (e.g., “I think the training was excellent and everything was, for the most part, perfectly straightforward” and “I think that the success program has been an amazing way to connect people who are in need of social support from people who understand long term concussions. It’s a great resource and should be available at every college”). Program recommendations included more support for setting goals with mentees (e.g., “I think recommendations for goals would be a good idea, maybe some things that people have chosen in the past.”), and one mentor reported that the weekly prompts were not useful in meeting specific mentee needs (“I think the program itself doesn’t leave room for a lot of individual differences between people. I found that trying to follow the program steps/suggestions for each meeting was difficult because my mentee did not feel the need to talk about some things that were suggested, and he wanted to talk about other things that weren’t really mentioned in the program.”).

Feedback about the app was particularly positive around having everything located in one place, and the general logic of the layout. For example, one mentor wrote, “The app is amazing! It is such a cool resource for a project like this. Educational handouts were easily accessible along with everything I needed to communicate with my mentee from the chat section to the handouts that I assigned and meetings I scheduled there”. However, other feedback highlighted problems with notifications (e.g., “I don’t always get notifications on the app so I find myself having to check it somewhat regularly so I don’t miss messages instead of just relying on a notification to tell me when I have a new message”), stability of the app (e.g., “The app also crashes sometimes when I’m trying to write a message to my mentee, so you have to rewrite messages a lot”), and lack of time zone support for the embedded calendars, all of which tempted mentors to share personal contact information to be able to use native mobile communication apps (“[My mentee] said she wasn’t receiving messages so I guess I wish that we could have texted or called outside of the app. That may have been easier.”).

Similar to mentee responses, mentors were enthusiastic about perceived psychosocial benefits of the intervention. One mentor commented, “I liked knowing that what I was saying was beneficial. There were lots of ‘lightbulb moments’ for him based on our conversations, and seeing his progress made me proud”. As others described, “It’s helpful getting advice, being connected to resources and having someone to share your thoughts with who understand and it’s rewarding helping someone through what was one of the most difficult points in your own life” and, “I think my favorite part of the program is still the opportunity to be there with/for someone on their concussion recovery journey. It’s nice to be able to share some of what I’ve learned on my own recovery path and have it help someone”. One mentor wrote about how their experience mirrored that of their mentee, writing, “I think it helped me to also know that I wasn’t alone in my experience”.

Some mentors shared other perceived benefits, such as how the program was motivating to their academics, supported them using strategies themselves, or helped them to reevaluate their injury in a more positive light. One mentor said, “I learned how much I like to help and to provide instruction to other people when I am knowledgeable on the topic. SUCCESS has made me prioritize my own work faster so that I have more time for my mentees. I love the program for me”. In the same vein, others wrote, “It helped me honestly stay on track with school and focus more”, “It also builds my interpersonal skills and helps me learn more about tailoring my communication and support to the unique needs of different people”, and “I think it helped me build my active listening skills. Also, it’s helped me to build self compassion—it’s much easier to take breaks while working after I’ve just spent half an hour helping talk someone else through why it’s important to take breaks”. One mentor described reframing their own relationship to their injury and seeing its value, “My concussion story shifted from being a funny story to tell at parties to an informative resource for other people and that was cool to see happen”. Mentors also described developing an understanding of their role and its value, and that this helped them build confidence (e.g., “At first I questioned what I could even offer her, since she was already doing ‘all the right things’. But by the end, I saw how I could be an effective mentor just by listening to what she was going through and that my perspective as someone who’s recovered from a concussion was still helpful. So I think I’m more confident now in my ability to help just by being with someone during their recovery and taking more of a passive approach.”).

## 4. Discussion

In the current study, postsecondary students with symptomatic concussions experienced improved symptoms, reduction of academic and mental health complaints, and gains in academic self-efficacy following an mHealth delivered peer mentoring intervention. Mentor measures of concussion symptoms, academics, and psychosocial health were stable from pre- to post-intervention. Educational materials about concussion, return to school and daily activities, and peer mentoring were provided in a mobile app, which also allowed for written and video communication between mentor and mentee dyads. Results from this preliminary investigation suggest that peer-led education and psychosocial support can be provided through an mHealth intervention and that students experiencing symptoms from concussion report improvement in daily function. Furthermore, providing mentoring is well-tolerated by students with previous concussions. 

### 4.1. Concussion, Academic, and Psychosocial Outcomes

Mentees experienced the reduction of concussion symptoms over the intervention period. Although improved symptoms are expected with acute concussion, mentees were an average of 3.6 months post-injury (median = 3), with even the most acute injury being greater than two weeks post-injury. Because about 70–80% of concussions in young adults are thought to resolve within month [7,9], this participant group likely reflects students at risk for protracted recovery or meeting criteria for persistent post-injury concussive symptoms [56]. Furthermore, the mentee participant group also reflected pre-injury factors known to complicate recovery, with 7 of the 16 having a previous history of concussion, and 12 being female, both factors associated with greater likelihood of prolonged symptoms [57,58]. In addition, mentees reported fewer academic disruptions as measured by the ADS at the post-mentoring timepoint. It is unclear whether those changes in academic function were independent of symptom changes, perhaps related to educational materials provided around academic supports and strategies, or in line with general symptom resolution. In the study that developed the ADS, most student complaints had resolved by 1 month after injury, with those who were female or had previous concussions being most likely to continue to report problems at that time [13]. Because the current group had similar risk factors, there may be some promise in the intervention having had an effect on academic function, but future studies would need to control for natural recovery and be large enough to conduct analyses more sensitive to change in symptoms versus academic activities. 

As expected, mentors experienced stable concussion symptoms and academics, but against our hypotheses, did not experience gains in psychosocial measures. Trends overall did align with anticipated outcomes, suggesting the current sample size was too small to observe such gains at a group level. On an individual level, most mentors did experience gains in self-efficacy and fewer mental health symptoms. Importantly, providing mentoring did not provoke previously resolved symptoms or cause undue stress or anxiety as mentors heard the concerns of their peers. No mentors expressed concerns about their own mental health, and a few reported that having this additional commitment in their schedule made them more organized, thus improving rather than stressing their academics. In addition, some mentors described that providing the educational handouts to their mentees and discussing how to use strategies to manage symptoms, schoolwork, and social life was a powerful reminder to themselves to do the same. As with other studies finding that mentoring serves not only the mentee but also the mentors [28,59,60], mentoring was well-tolerated and mentors reported appreciating the opportunity to reframe their own experience into having value by serving to support another person. 

### 4.2. Program Acceptability

Overall, mentee feedback indicated a high level of program acceptability. Levels of acceptability of the app were slightly lower, but notably, no mentees complained about the screentime involved in using an app or described their experience with the app as symptom provoking. In contrast, it appeared that using an app for communication with a mentor was very much in line with their daily social activities, and in this case, provided support relevant to their needs. That being said, mentors provided more elaborate feedback about the app functionality that likely explains lower ratings provided by mentees as well, namely, that notifications were inconsistently delivered and the app could at times crash unexpectedly. While mentees did not explicitly provide comments about the app functionality, the dyadic nature of the app means that these experiences were shared by mentees and were likely frustrating. Initial testing during development had resolved many complaints about the app, particularly stability of video calls [49], but overall moderate ratings of the app, as compared to the program, presents opportunities to continue to improve the app and its integration with the overall program design to strengthen it further [61]. Such a developmental cycle of testing followed by modification based on the target population feedback is consistent with principles of inclusive design which have been followed throughout SUCCESS program development [48,62].

In qualitative feedback provided post-intervention, mentees were particularly enthusiastic that the intervention was delivered by peers, who were viewed as knowledgeable confidants, in whom trust was quickly established. Education about concussion and recovery is known to positively affect outcomes [63], and a core intervention ingredient here is peer-provided education. Randomized controlled trials examining peer-led education for athletes have shown promise in improving knowledge and attitudes around reporting of injury [34]. Such peer-provided education is based on social learning theories, in which behaviors of in-group peers serve as models [20]. Although adults have been shown to benefit from such programming, postsecondary-aged emerging adults may be particularly primed to engage with peers in this stage of development [64]. Several other programs have demonstrated efficacy of peer mentoring for postsecondary students, improving academic outcomes and retention across a wide range of student groups [60,65,66,67,68]. Similarly, trials of peer-led educational interventions for people with disabilities show greater engagement in the intervention [29] when compared to traditional didactic models. When delivered by peers, even less formal educational delivery is associated with improved health and psychosocial outcomes [32]. The current results suggest that this likely extends to students with concussion, and aligns well with the needs of this group, for whom reassurance of recovery and social support are key components of care in neurobiopsychosocial models of concussion and recovery. Extended absences and removal from social groups are known to negatively affect student outcomes, especially in older students [69], so that embedding educational delivery in a social context, as was provided here, matches with student needs.

### 4.3. Limitations and Future Directions

Limitations to the current study should also be acknowledged. Multiple recruitment sites and methods were used, which likely resulted in heterogenous medical and rehabilitative experiences following injury. For example, some participants recruited through the specialty clinic may have also received rehabilitation services, such as vestibular therapy. However, by seeking care at a specialty clinic, those participants may have been experiencing more severe symptoms, or were at higher risk for complicated recovery. This pilot study was not designed to account for differences in care and rehabilitation, but future studies should examine the complementary role of peer support during versus after the conclusion of therapies. In addition, some resolution of symptoms of over time is expected and not accounted for in the current study design. Employing a waitlist or usual care control group for comparison would better allow for understanding of specific effects of the intervention. Qualitative analysis of participant experiences may also provide richer understanding of the utility of peer-mentoring for students recovering from concussion, particularly as symptom scales may not fully capture student perceptions of concussion recovery [70]. Lastly, future, larger studies should account for symptom severity and pre-injury complicating factors. 

Measurement of intervention dosage and adherence was also imprecise. Chat data were available, and mentoring pairs were instructed to meet weekly, but some may have met more or less frequently, as mentors often reported that finding a time that both students could meet was challenging. More refined reporting of metadata in real time to administrators may improve feedback to mentors, improving meeting adherence. In addition, the app does not currently support logging of meetings and meeting topics, which would allow for better understanding of intervention components and effects. Similarly, although the app was rated as being helpful, both mentees and mentors rated it as the least helpful of the program components. Future user-centered development work should describe organizational, communication, visual, or operation features that can be adapted to better meet the needs of this population of students.

The current sample included more females, consistent with increased risk for complicated concussion recovery among females [57], but may not fully capture the experiences of males or people with other gender identities. Follow-up research should target more balanced samples in both mentors and mentees, as well as provide greater racial and ethnic representation. Finally, several results approached but did not reach statistical significance, although visual display of individual results suggested directional trends among participants. A larger sample in future studies will provide greater clarity to evaluating this mobile health intervention and determine if improvements in self-efficacy and quality of life are observed as in other populations [32]. 

## 5. Conclusions

Mentoring has been used for peer-delivered education prior to concussion as well as psychosocial support for people with disabilities, but peer-mentoring as an intervention approach following concussion has been insufficiently explored. Based on the neurobiopsychosocial model of concussion, the peer mentoring approach directly targets many post-injury factors known to positively influence outcomes. Mobile health increases the reach of peer mentoring programs, reducing geographic and other barriers so that people can connect with others with similar experiences in a controlled virtual environment. In this preliminary study, postsecondary students with concussion—both mentors and mentees—reacted positively to an mHealth delivered peer mentoring intervention, and mentees demonstrated fewer symptoms, fewer academic complaints, and greater academic self-efficacy following mentoring. Mentor symptoms, academics, and mental health were unchanged, so the addition of mentoring responsibilities was well tolerated. Findings from this study underscore the importance of psychosocial support following concussion, and demonstrate that peer-led interventions can be delivered effectively to this population through mHealth. Postsecondary settings pose particular challenges around delivery of interventions to students. Results of the current study suggest that mHealth interventions facilitating just-in-time connections to peers and educational resources may be well suited for individuals with concussion. Training of mentors was also conducted virtually, increasing feasibility of program delivery. More research is needed to examine the impact of such peer-led psychosocial interventions following concussion, and to refine both the technology and delivery of interventions through mHealth platforms.

## Figures and Tables

**Table 1:** Participant Characteristics.

	Age	Gender	Race	Class Standing	Total Number of Concussions	Time Post Injury (Months)	Injury Mechanism	Program Chat Usage *
Mentee								
PR1	25	M	White	Graduate	1	5	Sports/Rec	28
PR2	22	F	White	Senior	1	4	MVC	77
PR3	21	F	Asian	Graduate	0	5	MVC	31
PR4	21	M	White	Senior	4	2.5	Struck By	43
PR5	26	F	White	Graduate	3	3	Struck By	29
PR6	20	F	White	Junior	1	1	Sports/Rec	49
PR7	25	F	White	Graduate	3	0.5	MVC	21
PR8	21	M	White	Junior	4+	2	Struck By	8
PR9	18	F	White	Freshman	2	3	Sports/Rec	25
PR10	21	F	White	Junior	1	12	Sports/Rec	27
PR11	19	F	White	Sophomore	3+	1	Sports/Rec	54
PR12	20	F	Black	Junior	1	7	Sports/Rec	10
PR13	21	F	Black	Junior	1	3	Struck By	12
PR14	20	F	Asian	Junior	1	7	Sports/Rec	18
PR15	19	F	Black	Sophomore	1	1	Struck By	31
PR16	20	M	White	Senior	2	2	Sports/Rec	23
Mentor								
MR1	23	F	White	Senior	1	21	Struck By	
MR2	20	F	Black	Junior	1	14	Struck By	
MR3	22	F	White	Senior	2	21	Sports/Rec	
MR4	22	M	Asian	New graduate (under-graduate)	3	7	MVC, Sports/Rec, Struck By	
MR5	20	F	White	Junior	2+	23	Sports/Rec	
MR6	21	NB	White	Senior	2+	31	Sports/Rec	
MR7	25	F	White	New graduate (under-graduate)	2+	24	Sports/Rec	
MR8	23	F	White	New graduate (under-graduate)	3	8	Sports/Rec	
MR9	23	F	White	Junior	0	24	MVC	
MR10	24	M	White	Senior	5	42	Sports/Rec	
MR11	21	F	White	Junior	0	12	MVC	

* Notes: F = female, M = male, NB = non-binary. Sports/rec = sports or recreation related injury. MVC = motor vehicle crash. Program chat usage reflects the total messages sent between a mentor and mentee.

**Table 2:** Summary of SUCCESS data sources.

Measurement Tools	Domain	Description
Post-Concussion Symptom Scale (PCSS) [51]	Concussion Symptoms	22 item self-report of presence and severity of concussion symptoms.
Academic Dysfunction Survey (ADS) [13]	Academics	29 item survey of self-reported academic problems post-concussion.
College and Academic Self-Efficacy Scale (CASES) [52]	Psychosocial Function: Academic Self-Efficacy	19 item self-report of confidence about completing academic tasks, and in likelihood of success as a student.
Patient Reported Outcome Measurement Information System (PROMIS) Self-Efficacy Instruments [53]	Psychosocial Function: General Self-Efficacy	10 item survey used to assess patient-reported health status for physical, mental, and social well–being.
Depression, Anxiety, Stress Scale (DASS) [54]	Psychosocial Function: Mental Health	42 item scale assessing mental health domains.
Traumatic Brain Injury Quality of Life Scale (TBI-QoL) [55]	Psychosocial Function: Quality of Life	10 item survey of participation in academic, vocational, and social activities (adapted to add academics).
Post-Mentoring Feedback Survey	Program and App Usage	Survey of program and app feedback with 8 open ended questions and 7 sliding scale (6 for mentors) questions asking participants to rank either the likelihood of recommending the program to a mentee or a mentor (2 questions) and helpfulness (5 questions for mentees, 4 for mentors) of the app or program features.
App Usage Data		Use of communication features (chat, videoconferencing) by frequency and type.

**Table 3:** SUCCESS Mentee Post-Mentoring Feedback Survey.

Item	Mean	Median	Range
How likely would you be to recommend this program to a friend to become a *Mentee* if they sustained a concussion? (*n* = 14) (0 = not at all likely; 100 = definitely recommend)	86.86	94	50–100
How likely would you be to recommend this program to a friend to become a *Mentor* if they had already recovered from a concussion? (*n* = 13) (0 = not at all likely; 100 = definitely recommend)	89.38	92	50–100
How helpful were each of the following? (0 = not at all; 100 = very helpful)			
Support provided by your Peer Mentor (*n* = 14)	96.07	100	77–100
SUCCESS Program Handouts (*n* = 11)	70	72	19–100
The App (*n* = 9)	62.89	60	17–100
The SUCCESS Team (*n* = 12)	81.08	80.5	52–100
Participating in the SUCCESS Program (*n* = 13)	90	96	56–100

**Table 4:** SUCCESS Mentor Post-Mentoring Feedback Survey.

Item	Mean	Median	Range
How likely would you be to recommend this program to a friend to become a Mentee if they sustained a concussion? (0 = not at all likely; 100 = definitely)	96.6	100	75–100
How likely would you be to recommend this program to a friend to become a Mentor if they had already recovered from a concussion? (0 = not at all likely; 100 = definitely)	93.7	100	75–100
How helpful were each of the following? (0 = not at all; 100 = very helpful)			
SUCCESS training	87.5	93	50–100
SUCCESS Program Handouts	78.7	78.5	50–100
The App	76.3	90	26–100
The SUCCESS Team	99.2	100	97–100

## Data Availability

The raw data supporting the conclusions of this article will be made available by the authors, without undue reservation.

## Supplementary Materials

The following supporting information can be downloaded at: https://www.mdpi.com/article/10.3390/ijerph20085438/s1, Figure S1: Summary of SUCCESS Infographic Educational Handouts; Table S1: SUCCESS Mentor Training Outlines.
