# Supplementary material for: The SUCCESS Peer Mentoring Program for College Students with Concussion: Preliminary Results of a Mobile Technology Delivered Intervention
Source: Int J Environ Res Public Health. 2023 Apr 7;20(8):5438. doi: 10.3390/ijerph20085438 (PMC10138278; doi:10.3390/ijerph20085438)
Supplement: Supplementary file 1 [file ijerph-20-05438-s001.zip › ijerph-2237086-supplementary.pdf]

The SUCCESS Peer Mentoring Program for College Students with Concussion: Preliminary Results of a Mobile Technology Delivered Intervention

Katy H. O'Brien, Yalian Pei, Amy M. Kemp, Rebecca Gartell, Russell K. Gore, and Tracey Wallace

Table S1. SUCCESS Mentor Training Outlines

**SUCCESS Mentor Training Part 1 – Mentoring and Program Training Outline**	
Introduction and Overview	1. Introduction<br>a. Overview of the SUCCESS Peer Mentor Training<br>b. Completing this Training<br>2. What is SUCCESS?<br>a. Why Peer Mentoring?<br>b. What is the SUCCESS program?<br>i. Concussion basics<br>ii. Return to Learn 1 – Advocacy and Accommodations<br>iii. Return to Learn 2 – Strategies and Resources<br>iv. Return to Life<br>3. Meet the SUCCESS Team<br>4. Why Peer Support (video)<br>5. Peer Mentoring Relationships and Roles<br>6. Knowledge Check 1: SUCCESS and Peer Support (closed-ended questions)<br>7. Program Component Overview<br>8. Dos and Don'ts for Peer Mentors<br>9. Ethical Peer Mentoring<br>10. Knowledge Check 2: The Role of Peer Support (closed-ended questions)<br>11. Create Your Mentoring Profile<br>a. About You (open and closed-ended questions)<br>b. About Your Experience with Concussion (open and closed-ended questions)<br>c. Self-Reflection: Why Do You Want to be a Peer Mentor? (open-ended question)
Unit 1: Understanding Concussion	1. What is a Concussion Anyway?<br>2. Concussion Recovery<br>3. Concussion Symptoms<br>4. Before You Had One, What Did You Know About Concussion? (video)<br>5. Self-Reflection 1.1: Your Concussion Knowledge Experience (open-ended questions)<br>6. What Does a Concussion Mean for College? (video)<br>7. Concussion and College (video)<br>8. Self-Reflection 1.2: Your Challenges (open-ended questions)<br>9. Strategies to Recover and Be Successful in School (video)<br>10. Self-Reflection 1.3: Your Strategies (open-ended questions)

Unit 2: Understanding Peer Coping	1. Why are Peer Mentors Important for Coping? (video)<br>2. Coping and Stress<br>3. Emotion-Focused Coping<br>4. Problem-Focused Coping<br>5. What is Empathy? (video)<br>6. Knowledge Check 2.1: What Role Does Empathy Play in Peer Coping? (closed-ended questions)<br>7. Self-Reflection 2.1: Your Coping Strategies (open-ended questions)<br>8. Applying Emotion and Problem-Focused Coping (video)<br>9. Self-Reflection 2.2: Your Coping Needs (open-ended questions)<br>10. Self-Reflection 2.3: Your Coping Experience (open-ended questions)
Unit 3: Building Communication Skills	1. Effective Communication Skills (video; open-ended question)<br>2. Active Listening<br>3. Communication Skills: The OARS Model of Communication<br>a. O = Open Questions<br>b. A = Affirmations<br>c. R = Reflective Listening<br>d. S = Summarizing<br>4. Scenario 3.1: Active Listening and OARS (open and closed-ended questions)<br>5. Scenario 3.2: Active Listening and OARS (open and closed-ended questions)<br>6. Scenario 3.3: Active Listening and OARS (open and closed-ended questions)<br>7. Scenario 3.4: Active Listening and OARS (open and closed-ended questions)<br>8. Self-Reflection 3.1: Effective Communication
Unit 4: Encouraging Self-Advocacy and Finding Resources	1. What is Self-Advocacy and Why Does it Matter? (video)<br>2. Being a Proactive Self-Advocate<br>3. Communicating Needs<br>4. Communicating Needs with I-Statements<br>5. Campus Resources and Student Rights<br>6. Self-Reflection 4.1: Your Self-Advocacy Experience (open and closed-ended questions)<br>7. Knowledge Check 4.1: Self-Advocacy Skills (open and closed-ended questions)
Unit 5: Using Your Team	1. Finding Balance in Peer Mentoring<br>2. Being Part of the Team<br>3. Knowledge Check 5.1: Being Part of the Team (open and closed-ended questions)<br>4. Promoting Healthy Behaviors and Choices

	5. Knowledge Check 5.2: Promoting Healthy Behaviors and Choices (video; open and closed-ended questions)<br>6. Recognizing Red Flags and Warning Signs<br>7. Warning Signs – What to Look For<br>8. Recognizing Warning Signs<br>9. What to do with Warning Signs – Yellow Flag Action Plan<br>10. What to do with Warning Signs – Red Flag Action Plan<br>11. Responding to Warning Signs<br>12. Knowledge Check 5.3: Reacting to Warning Signs (open and closed-ended questions)<br>13. Knowledge Check 5.4: Reacting to Warning Signs (open and closed-ended questions)
Thank You and Next Steps	

**SUCCESS Mentor Training Part 2 – Program and App Training Outline**	
Introduction and Purpose	
Unit 1: Overview of the SUCCESS Mentoring App	1. Getting to Know the SUCCESS App<br>a. Getting Started: Setting Up Your Profile (video)<br>b. Technology Check 1.1 (closed-ended question)<br>2. About Matching with a Mentee<br>3. Exploring Other Features of the App (video)<br>4. Your "Me" Page (video)<br>5. Getting to Know Your Mentee (video)<br>6. Knowledge Check 1.1: You've Been Matched (closed-ended questions)
Unit 2: Getting to Know the SUCCESS Learning Resources	1. The SUCCESS Learning Resources<br>2. Learning Resource Topics<br>3. Knowledge Check 2.1 (closed-ended questions)
Unit 3: Connecting with Your Mentee	1. Mentoring Your Mentee: A Quick Guide<br>2. Meeting Your Mentee<br>3. Using the App to "Meet" Your Mentee (video)<br>4. Interacting in the App (video)<br>5. Logging Meetings (video)<br>6. The SUCCESS Web Portal (video)<br>7. Communication Tips<br>a. First Meeting<br>b. Subsequent Meetings<br>8. Mentor Communication Tips

	9. Closing Out the Mentor and Mentee Relationship<br>10. Phases of the Mentoring Relationship<br>11. Connecting Outside of SUCCESS<br>12. Knowledge Check 3.1 (closed-ended questions)
Unit 4: Contacting Us	1. When to Reach Out to the SUCCESS Team<br>2. Contacting Us<br>3. Knowledge Check 4.1 (closed-ended questions)
Unit 5: Readiness for SUCCESS	1. Readiness for SUCCESS (open and closed-ended questions)
Final Comments and Thank You	

Figure S1. Summary of SUCCESS Infographic Educational Handouts.

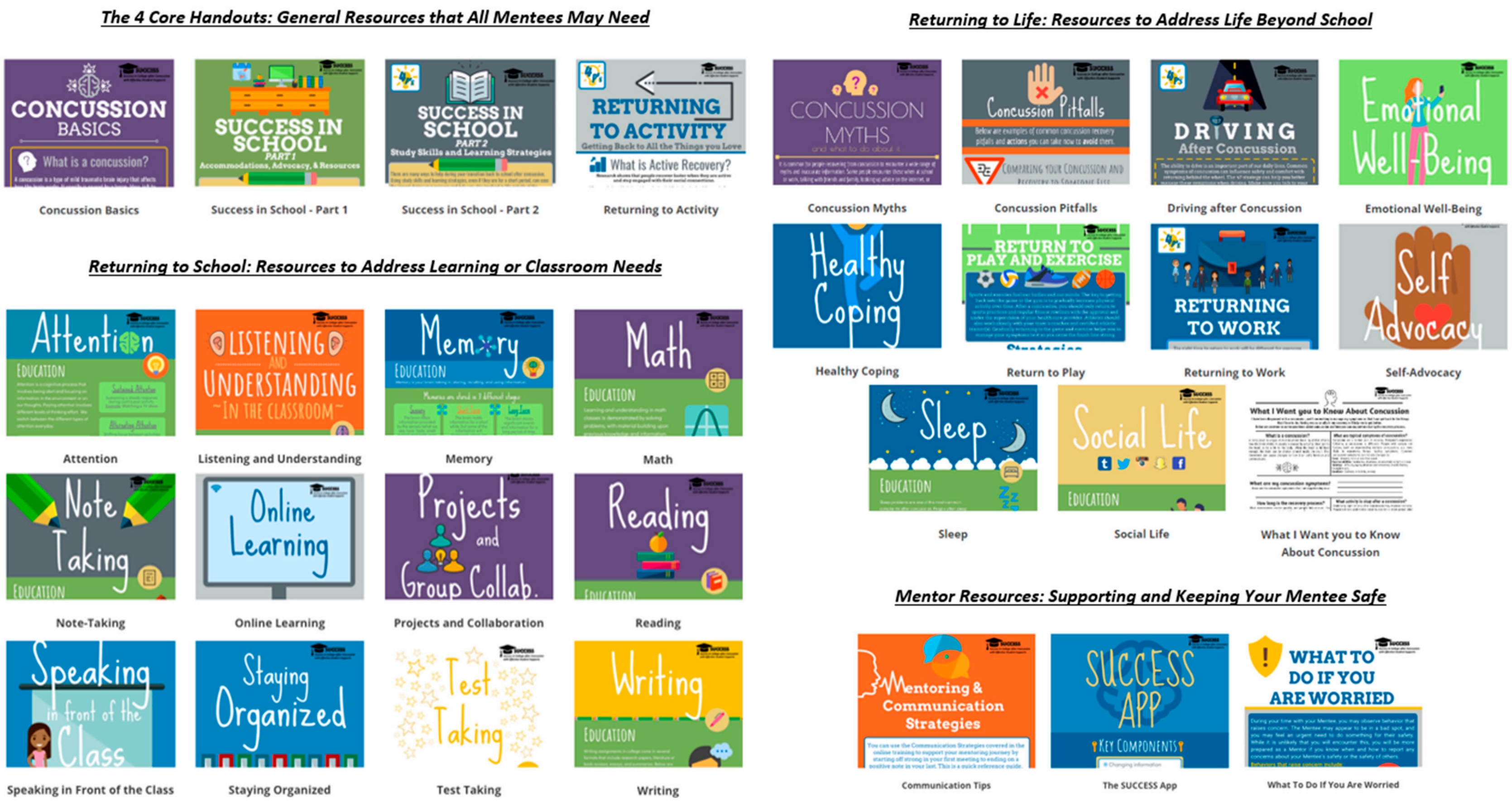

All handouts are available for download at: http://www.peersuccess.org/education.html
